# Tetramethylpyrazine alleviates hypoxia-induced proliferation, migration, and inflammatory response of fibroblast-like synoviocytes via inhibiting the HIF-1α- circCDC42BPB pathway

**DOI:** 10.1186/s42358-024-00355-1

**Published:** 2024-03-06

**Authors:** Yu-jing Zhang, Li-feng Chen, Xu Li, Jian-hua Chen, Zhang-kui Tan

**Affiliations:** 1 Department of Rheumatology, General Hospital of Central Theater Command, No. 627 Wuyi Road, Wuchang District, Wuhan, Hubei 430070 China; 2 Department of Cardiology, Guiqian International General Hospital, No. 1 Dongfeng Avenue, Wudang District, Guiyang, Guizhou 550018 China

**Keywords:** circCDC42BPB, HIF-1α, RA-FLSs, Hypoxia

## Abstract

**Objectives:**

Rheumatoid arthritis (RA) is a chronic inflammatory joint disease, which might trigger cartilage, bone damage, and disability. Recent studies have suggested that Tetramethylpyrazine (TMP), an alkaloid monomer isolated from the rhizome of the traditional herbal medicine Ligusticum wallichii Franch, exerts a broad spectrum of pharmacological properties, containing anti-inflammatory. This study aimed to analyze the role and underlying mechanism of TMP in RA.

**Methods:**

Under Hypoxia condition, RA-Fibroblast-like synoviocyte (FLS) were treated with TMP at different doses. Cell viability, proliferation, cell cycle progression, and migration were detected using Cell Counting Kit-8 (CCK-8) assay, 5-ethynyl-2’-deoxyuridine (EdU) assay, flow cytometry assay, wound healing assay, and transwell assay. Cyclin D1, Proliferating cell nuclear antigen (PCNA), Matrix metalloproteinase-2 (MMP2), MMP9, and hypoxia-inducible factor-1α (HIF-1α) protein levels were measured using western blot assay. Interleukin-6 (IL-6) and IL-8 were evaluated using ELISA. Circular RNA (circRNA) hsa_circ_0005178 (circCDC42BPB), CDC42BPB, and HIF-1α expression were determined using real-time quantitative polymerase chain reaction (RT-qPCR). Binding between HIF-1α and CDC42BPB promoter was predicted by JASPAR and verified using dual-luciferase reporter and Chromatin immunoprecipitation (ChIP) assays.

**Results:**

TMP might hinder FLS proliferation, cycle progression, migration, and inflammatory response under hypoxic conditions. CircCDC42BPB expression was increased in RA patients and RA-FLSs treated with hypoxia, while its level was obviously reduced in RA-FLSs treated with hypoxia and TMP. TMP might abolish hypoxia-induced circCDC42BPB expression. Upregulation of circCDC42BPB might partially overturn the repression of TMP on hypoxia-caused RA-FLS damage. TMP might regulate circCDC42BPB level via HIF-1α in RA-FLSs under hypoxic conditions.

**Conclusion:**

TMP might block RA-FLS injury partly via regulating the HIF-1α- circCDC42BPB pathway, providing a promising therapeutic target for RA.

**Supplementary Information:**

The online version contains supplementary material available at 10.1186/s42358-024-00355-1.

## Highlights

• TMP suppressed hypoxia-induced RA-FLS growth and inflammatory response.

• TMP might repress circCDC42BPB expression in RA-FLSs under hypoxic conditions.

• TMP might inhibit HIF-1α-induced circCDC42BPB transcription under hypoxic conditions.

## Introduction

As the most frequent chronic inflammatory joint disease characterized by synovial inflammation and joint damage, Rheumatoid arthritis (RA) has been recognized as a major public health challenge all over the world [1]. Clinically, this disease characteristically involves musculoskeletal pain, swelling, and stiffness, which ultimately lead to disability and even reduced life expectancy [2]. The precise cause of RA remains unknown, but there are clearly genetic and environmental influences involved [3, 4]. Notably, current studies suggested that fibroblast-like synoviocytes (FLSs) are vital effector cells in the pathogenesis of RA [5]. In the synovial lining, FLSs play an essential part via the generating of cytokines that perpetuate inflammation and proteases that promote cartilage destruction [6]. In the pathological RA state, activated RA-FLSs represent a significant driver of synovial inflammation and joint damage, exhibiting an aggressive tumor-like phenotype [7]. Therefore, it is believed that the regulation of aggressive behavior in FLSs is an important strategy in RA management.

During the past decades, increasing attention has been paid to the use of traditional Chinese medicine (TCM) in several diseases, containing RA [8]. As an alkaloid monomer that exists in the rhizome of the traditional herbal medicine Ligusticum wallichii Franch, Tetramethylpyrazine (TMP), has been identified to possess a broad spectrum of pharmacological properties [9], containing anti-oxidant, anti-inflammatory, and anti-fibrosis. Currently, some laboratory studies have shown that TMP treatment might alleviate the progression of different diseases, such as alcoholic liver disease [10], ischemic cardiovascular disease [11], and Alzheimer’s disease [12]. Furthermore, some recent animal experiments have indicated that TMP might protect against multiple disorders, including arthritis treatment, by repressing inflammation and oxidative stress [13, 14]. Nevertheless, the mechanism by which TMP alleviates RA is far from being addressed.

Pervasive transcription of the human genome has produced a large number of non-coding RNAs, most of which play vital regulatory roles in shaping cellular activity [15]. As a naturally occurring family of non-coding RNA molecules formed by back-splicing events, circular RNAs (circRNAs) have a stable closed continuous loop structure with neither 5’ to 3’ polarity nor polyadenylated tail [16, 17]. This structural characteristic has conferred on them inherent resistance to exonucleolytic RNA decay, which therefore makes them more stable than traditional linear RNA [18]. Moreover, the superior features of circRNAs have been well established, such as high abundance, evolutionary conserved, and cell type-specific expression manner, which imply that they are promising as biomarkers for diverse diseases [19]. Previous studies have shown that an important part of circRNA function is attributed to the interaction with RBPs, which are a large category of proteins implicated in gene transcription and translation [20, 21]. Growing evidence shows that some circRNAs are differentially expressed in RA patients and serve as essential regulators in RA progression [22, 23]. Herein, hsa_circ_0005178 (circCDC42BPB) was identified to be aberrantly upregulated in RA subjects, but whether it is involved in the modulation of TMP is unknown in the RA process.

Work in several laboratories has revealed that the hypoxic microenvironment is one of the most essential characteristics of RA, primarily due to synovial expansion outstrips the oxygen supply, resulting in areas of synovial hypoxia [24–26]. Meanwhile, chronic hypoxia could be responsible for persistent synovitis and erosion of cartilage and bone [27]. At the cellular level, it boosts synovial cell viability and migration in RA via changing signaling pathways and gene expression [28]. Notably, the cellular response to hypoxia is major driven by the transcription factor hypoxia-inducible factor-1α (HIF-1α), which is widely expressed in rheumatoid synovial tissue and synoviocytes [29, 30]. Of interest, HIF-1α might transcriptionally modulate target gene expression via combining with the hypoxia response elements (HREs) in their promoter regions, which in turn affects the development and progression of human tumors [31, 32]. Yet, whether HIF-1α partake in regulating circCDC42BPB transcription is an unelucidated issue in RA.

In the present study, our results demonstrated that applying TMP might repress hypoxia-induced RA-FLS damage in a concentration-dependent manner. CircCDC42BPB expression was improved in RA patients and RA-FLSs treated with hypoxia, while its content was significantly decreased in RA-FLSs treated with hypoxia and TMP. Furthermore, TMP might relieve hypoxia-induced circCDC42BPB expression. Apart from that, HIF-1α might facilitate the biogenesis of circCDC42BPB. Our findings suggested that TMP might be involved in hypoxia-induced RA-FLS damage via HIF-1α/circCDC42BPB.

## Materials and methods

### Serum samples and cell culture

Peripheral blood samples were collected from each RA sufferer (*n* = 50) and each healthy volunteer (*n* = 25) at the General Hospital of Central Theater Command in this research, which was approved by the Ethics Committee of General Hospital of Central Theater Command. After centrifugation, the serum specimens were acquired. Meanwhile, all participants signed written informed consent prior to enrolling in this study. The detailed clinical characteristics of patients and healthy volunteers are described in Table 1.

**Table 1:** The clinical characteristics of the patients with RA and Normal

	Normal(n = 25)	RA(n = 50)	*P* value
Gender			0.402
Males	17	29	
Females	8	21	
Age (years)	45.61 ± 5.62	47.24 ± 9.12	0.408
DAS28	/	4.38 ± 1.29	
CRP (mg/L)	/	24.57 ± 6.85	
ESR (mm/h)	/	29.63 ± 13.14	
RF (IU/ml)	/	34.29 ± 8.46	

Measurement data were expressed as mean土SD. The unpaired t test was used for comparisons between two groups

Measurement data were expressed as the number of cases, and Fisher’s exact test was used for comparatives between groups

RA: Rheumatoid arthritis; DAS28: Disease activity score; CRP: C-reactive protein; ESR: Erythrocyte sedimentation rate; RF: Rheumatoid factor

In addition, RA-FLSs were isolated as described previously [33, 34]. Briefly, synovial tissues from RA sufferers (same as blood donors) were obtained during knee joint arthroscopy and immediately placed in RPMI1640 medium (Sigma-Aldrich, St. Louis, MO, USA). After being minced into about 5 mm pieces, these tissues with the medium were evenly spread on the bottom of cell culture flasks for 6 h at 37˚C, followed by the addition of 10% FBS and 1% penicillin/streptomycin. Subsequently, the non-adherent tissue pieces were taken out via changing the culture medium every 3 ~ 5 days. Then, the primary synovial cells were allowed to reach 70 ~ 80% for passaging. Finally, FLSs from passages 4 to 8 were applied in the following assays. For hypoxic experiments, RA-FLSs were exposed to 2% O_2_ in a hypoxia incubator chamber (ASTEC, Fukuoka, Japan) for 24 h as a hypoxia group. Meanwhile, these RA-FLSs were induced with 0, 10, 20, and 40 µM of TMP (Sigma-Aldrich) for 24 h. Besides, 40 µM TMP treatment for 24 h was used for further functional experiments. Besides, cells were incubated under normoxic conditions in a 5% CO_2_ incubator for 24 h as a normoxic group.

### Counting kit (CCK-8) assay

Assessment of RA-FLS viability was performed in this experiment. In short, treated or untreated 5 × 10^3^ cells under hypoxic conditions were cultured overnight. At indicated time points, CCK-8 (10 µL, Dojindo, Kumamoto, Japan) was allowed to be added into each well. A further 4 h later, a microplate reader at 450 nm was applied to record the absorbance.

### 5-ethynyl-2’-deoxyuridine (EdU)

Generally, 1 × 10^4^ RA-FLSs were grown for 48 h in six-well plates, followed by incubation with 50 µM EdU working solution (RiboBio, Guangzhou, China). 2 h later, 4% formaldehyde was utilized to fasten the cells, which then were reacted with Apollo reaction cocktail and DAPI for 30 min. Finally, samples were visualized based on a fluorescence microscope.

### Flow cytometry for cell cycle progression

After harvesting and washing in ice-cold PBS, 70% ethanol at 4˚C was used to fix 1 × 10^5^ RA-FLSs, which then were centrifuged and mixed with RNase (50 µg/mL) for 30 min. Then, nucleic acids were stained with Propidium Iodide (PI, Bender Med System, Vienna, Austria). At last, samples were processed using a FACSCalibur Flow Cytometer (BD Biosciences, Franklin Lakes, NJ, USA) and Cell Quest Pro. Results were analyzed using ModFit 3.1 software (BD Biosciences).

### Western blot assay

In brief, whole cell lysates from treated RA-FLSs were generated using RIPA lysis buffer (Keygen, Nanjing, China) containing protease inhibitors. After separation on the gel, the proteins were loaded onto nitrocellulose membranes (Sigma-Aldrich), which then were incubated overnight at 4˚C, with primary antibodies: Cyclin D1 (1:200, ab16663, Abcam, Cambridge, MA, USA), Proliferating cell nuclear antigen (PCNA, 1:1000, ab29, Abcam), MMP2 (1:1000, ab86607, Abcam), MMP9 (1:1000, ab76003, Abcam), HIF-1α (1:2500, 20960-1-AP, Proteintech Group, Rosemont, IL, USA), and GAPDH (1:50000, 60004-1-lg, Proteintech Group). At last, the ECL reagent (Millipore, Molsheim, France) was utilized to visualize the bands after incubation with a secondary antibody for 2 h.

### Cell migration assay

Measurement of RA-FLS migration ability was carried out using wound healing and transwell assays. Briefly, 1 × 10^5^ treated RA-FLSs in 6-well plates were cultured overnight to generate the monolayer confluence. Then, a sterile pipette tip was applied to make standardized wound scratches (time 0 h), followed by replacement with serum-free medium. After that, cells were allowed to migrate for 24 h, and the gap size was captured and analyzed using Image J software. In terms of transwell assay, transwell chambers (BD Biosciences) were introduced with 5 × 10^4^ RA-FLSs with serum-free medium, while the lower compartment possessed a complete medium. After a 24 h incubation, the cells attached to the lower surface were fixed and stained, followed by microscope observation.

### Enzyme-linked immunosorbent assay (ELISA)

After multiple treatments, RA-FLS supernatants were collected from cell culture experiments, followed by measurement of IL-6 and IL-8 levels using specific ELISA kits (BMS213-2, KHC0081, Invitrogen, Paisley Scotland, UK).

### Real-time quantitative polymerase chain reaction (RT-qPCR)

For RA-FLS RNA extraction, Trizol reagent (Invitrogen) was utilized. After synthesizing temple DNA using High-Capacity cDNA Reverse Transcription Kit (Applied Biosystems, Foster City, CA, USA), amplification reaction was conducted on Thermal Cycler CFX6 System (Bio-Rad, Hercules, CA, USA) with SYBR Green PCR Kit (Takara, Tokyo, Japan). Relative expression was calculated by the 2^–ΔΔCt^ method, normalizing to GAPDH. Primer sequences were presented in Table 2.

**Table 2:** Primers sequences used for PCR

Name		Primers for PCR (5’-3’)
hsa_circ_0004912	Forward	AGCGTGCATTATTTCTGTCTTCTG
	Reverse	TTACAGTCTTCATTCGGTGCTG
hsa_circ_0005178 circCDC42BPB	Forward	TTACGTGCACAGCTAAACCA
	Reverse	AACCTCACCAAAAGCACCTCT
hsa_circ_0092297	Forward	GCAGCAGGTGTCTTGCATCT
	Reverse	TCCCATACAAACAGCCCACTC
hsa_circ_0007514	Forward	CCGGAGGGATTCCATCATAGAG
	Reverse	GCGGCCGTGCTACAGG
hsa_circ_0047663	Forward	TTGGCAGGTGTGCGCAGAAC
	Reverse	TAACGTCGGAATGGTACACACTG
CDC42BPB	Forward	GACAAGTACGTGGCCGAGTT
	Reverse	GCAACCTCACCAAAAGCACCT
HIF-1α	Forward	CGGCGCGAACGACAAGAAAA
	Reverse	GAAGTGGCAACTGATGAGCA
GAPDH	Forward	TCGGAGTCAACGGATTTGGT
	Reverse	TTCCCGTTCTCAGCCTTGAC

For subcellular fractionation assay, RNA isolation of nuclear and cytoplasmic fractions from RA-FLSs was performed using PARIS Kit (Ambion, Austin, TX, USA). Then, circCDC42BPB, U6 (nucleus control), and GAPDH (cytoplasm control) expression levels were determined using RT-qPCR.

To validate the circular feature of circCDC42BPB, RNase R (Seebio, Shanghai, China) was applied for the digestion of total RNAs from RA-FLSs. At 20 min after incubation at 37˚C, the abundance of circCDC42BPB and liner CDC42 binding protein kinase beta (CDC42BPB) were measured using RT-qPCR.

### Cell transfection

For circCDC42BPB overexpressing (OE-circCDC42BPB), the full-length circCDC42BPB (without intervening introns) sequence was synthesized and cloned into a lentiviral GV248 vector (Genechem, Shanghai, China). Lentiviral GV248 empty vector performed as negative control (vector). Subsequently, the construct and lentivirus packaging plasmids (psPAX2 and pMD2.G) were co-transfected into 293T cells. After being filtered and enriched, these Lentiviral particles were transduced into RA-FLSs at 60% confluency in the presence of 8 µg/mL polybrene. At last, 5 µg/mL puromycin was used to select stably expressing cell lines.

Meanwhile, HIF-1α small interfering RNA si-HIF-1α: 5’-AACUUAUCUUUUUCUUGUCGU-3’ (sense), 5’-GACAAGAAAAAGAUAAGUUCU-3’(antisense) or its control (si-NC) from RiboBio (Guangzhou, China) and the plasmids: pcDNA and pcDNA- HIF-1α (HIF-1α, NM_001530.4) were introduced into RA-FLSs using Lipofectamine 3000 (Invitrogen) for 48 h.

### Dual-luciferase reporter assay

This experiment was performed as previously described [31]. For assessing circCDC42BPB promoter activity, the promoter (-2000 to -1 bp) sequences of CDC42BPB possessing the wild-type (WT) or mutant-type (MUT) HIF1α binding sites (RiboBio) were introduced into dual-luciferase reporter vector GV-238 (Genechem). Subsequently, these vectors were co-transfected into RA-FLSs along with si-HIF1α or si-NC for 48 h, followed by the analysis of luciferase activity using Dual Luciferase Assay Kit (Promega, Madison, WI, USA). Luciferase reporter assay was performed at least three times.

### Chromatin immunoprecipitation (ChIP)

ChIP kit (Wanleibio, Liaoning, China) was employed for this experiment. After being cross-linked with 1% formaldehyde, RA-FLSs were added with glycine to terminate this reaction. After that, the DNA fragments were mixed with anti-HIF1α (Novus Biologicals, CO, USA), anti-IgG, and anti-Polymerase II (Pol II, Wanleibio). 16 h later, these products were subjected to RT-qPCR analysis.

### Statistical analysis

In this research, data comparison was processed using Student’s *t*-test or one-way analysis of variance (ANOVA) with Tukey’s tests. GraphPad Prism7 software was used to analyze the statistical difference results, which were displayed as mean ± standard deviation (SD). *P*-value < 0.05 was applied as the threshold of significance.

## Results

### TMP might repress hypoxia-induced RA-FLS proliferation, migration, and inflammatory response

**Fig. 1:**
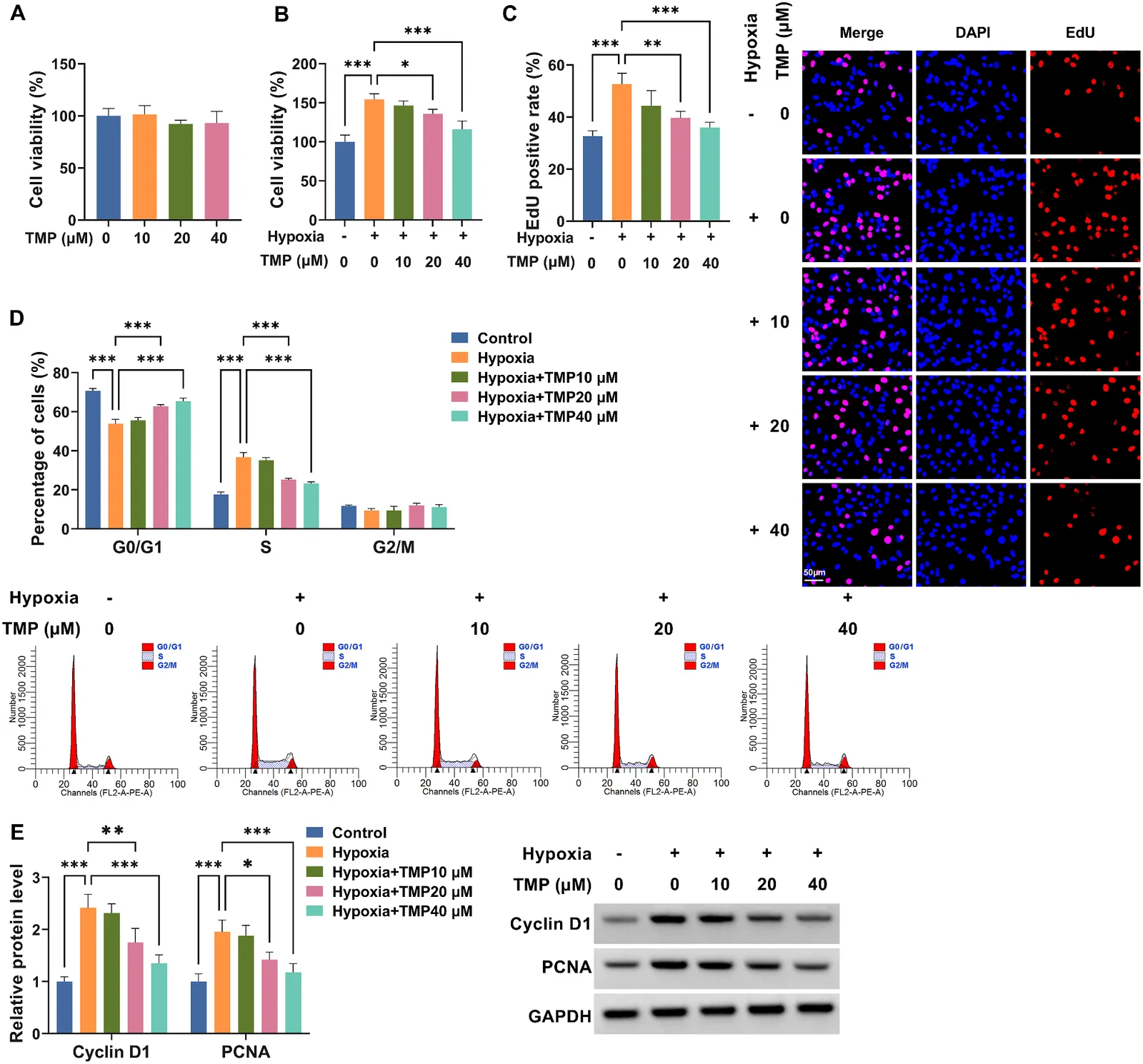
Effects of TMP on RA-FLS proliferation under hypoxic conditions. RA-FLSs were exposed to hypoxic conditions for 24 h in the presence or absence of TMP (0, 10, 20, and 40 µM). (**A**) Cell viability was assessed in RA-FLS treated with TMP (0, 10, 20, and 40 µM) using CCK-8 assay (one-way ANOVA). (**B** and **C**) Cell viability and proliferation were assessed using CCK-8 and EdU assays (one-way ANOVA). (**D**) Cell cycle progression was examined using flow cytometry assay (one-way ANOVA). (**E**) Protein levels of Cyclin D1 and PCNA were measured using western blot assay (one-way ANOVA). *P < 0.05, **P < 0.01, ***P < 0.001, *n* = 3

**Fig. 2:**
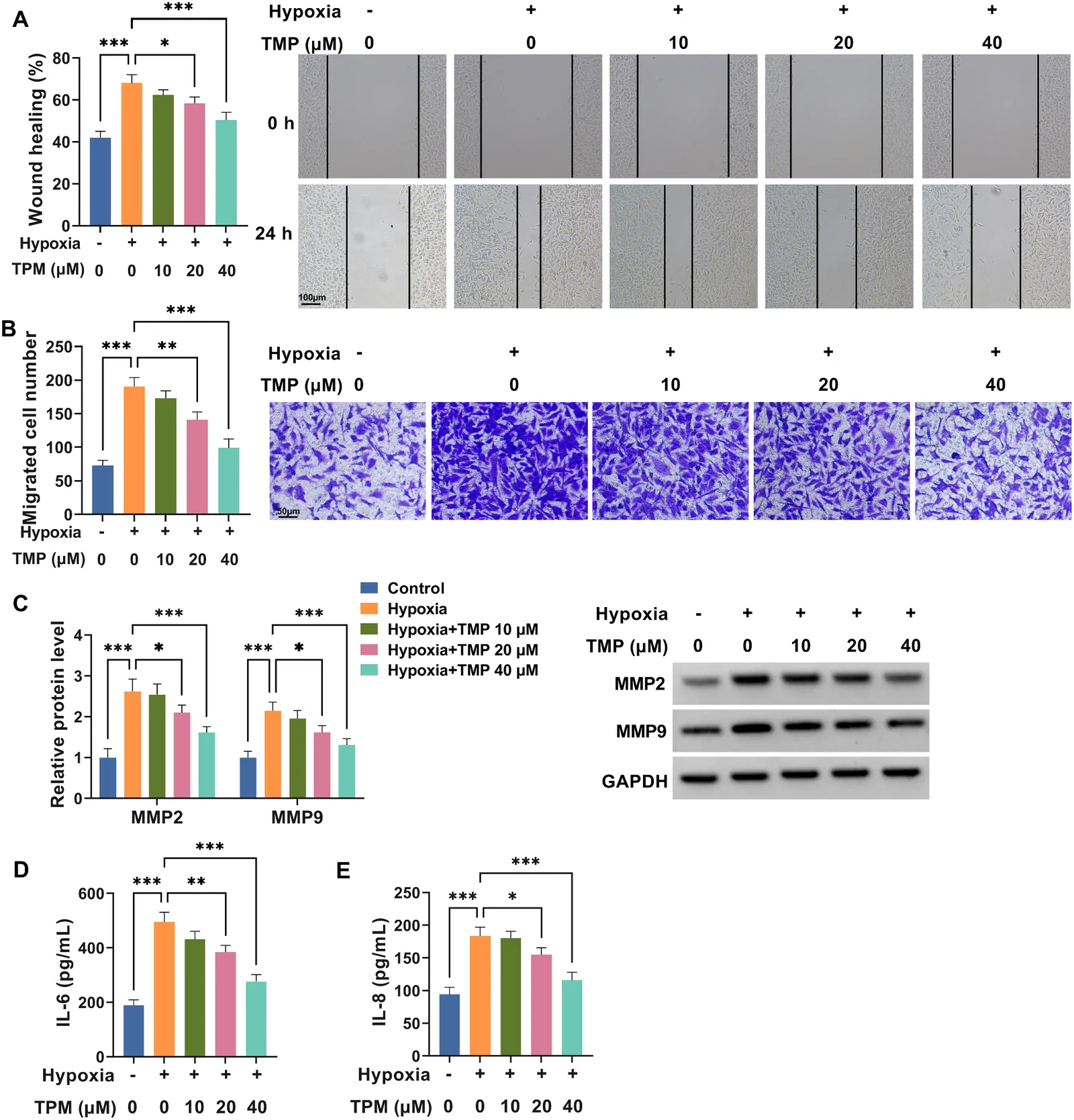
Effects of TMP on RA-FLS migration and inflammatory response under hypoxic conditions. RA-FLSs were treated with hypoxic conditions for 24 h in the presence or absence of TMP. (**A** and **B**) Wound healing and transwell assays were used to detect RA-FLS migration (one-way ANOVA). (**C**) Western blot assay was performed to determine the protein levels of MMP2 and MMP9 (one-way ANOVA). (**D**) Concentration of cytokines (IL-6 and IL-8) in RA-FLS cells culture medium of different groups was measured by ELISA Kits (one-way ANOVA). *P < 0.05, **P < 0.01, ***P < 0.001, *n* = 3

Initially, CCK-8 assay suggested that TMP treatment has no effect on RA-FLS viability (Figure A). Then, to check the direct cytotoxic influence of TMP on human RA-FLSs under hypoxic conditions, RA-FLSs were treated with different concentrations of TMP (0, 10, 20, and 40 µM) for 24 h. As shown in Fig. 1B, cell viability was gradually reduced in RA-FLSs after the treatment of 0–40 µM TMP. Subsequently, under hypoxic conditions, EdU results displayed that RA-FLS proliferation was also apparently decreased in a dose-dependent manner upon TMP treatment (Fig. 1C). Apart from that, flow cytometry assay exhibited that the percentage of RA-FLS in the G0/G1 phase was significantly enhanced and the percentage of cells in the S phase was evidently reduced after TMP exposure in a concentration-dependent way (Fig. 1D), implying the repression of TMP treatment on cycle progression. Meanwhile, western blot assay presented that Cyclin D1 (cell cycle regulator) and PCNA (cell proliferation marker) protein levels were also clearly hindered by TMP treatment (Fig. 1E). Beyond that, wound healing and transwell analysis presented that applying TMP might significantly relieve hypoxia-stimulated RA-FLS migration enhancement in a concentration-dependent way (Fig. 2A and B). Meanwhile, western blot assay exhibited that MMP2 and MMP9 protein levels (migration-related factors) in hypoxic conditions were also obviously downregulated in response to TMP in RA-FLSs in a dose-dependent manner (Fig. 2C). In terms of the inflammatory response, sections of pro-inflammatory cytokines IL-6 and IL-8 were highly triggered under hypoxic conditions, which was overturned by TMP treatment in a concentration-dependent manner (Fig. 2D). Together, these data suggested that applying TMP might ameliorate hypoxia-caused RA-FLS injury, and 40 µM TMP treatment for 24 h was chosen for further functional analysis.

### TMP treatment might suppress hypoxia-induced circCDC42BPB expression

**Fig. 3:**
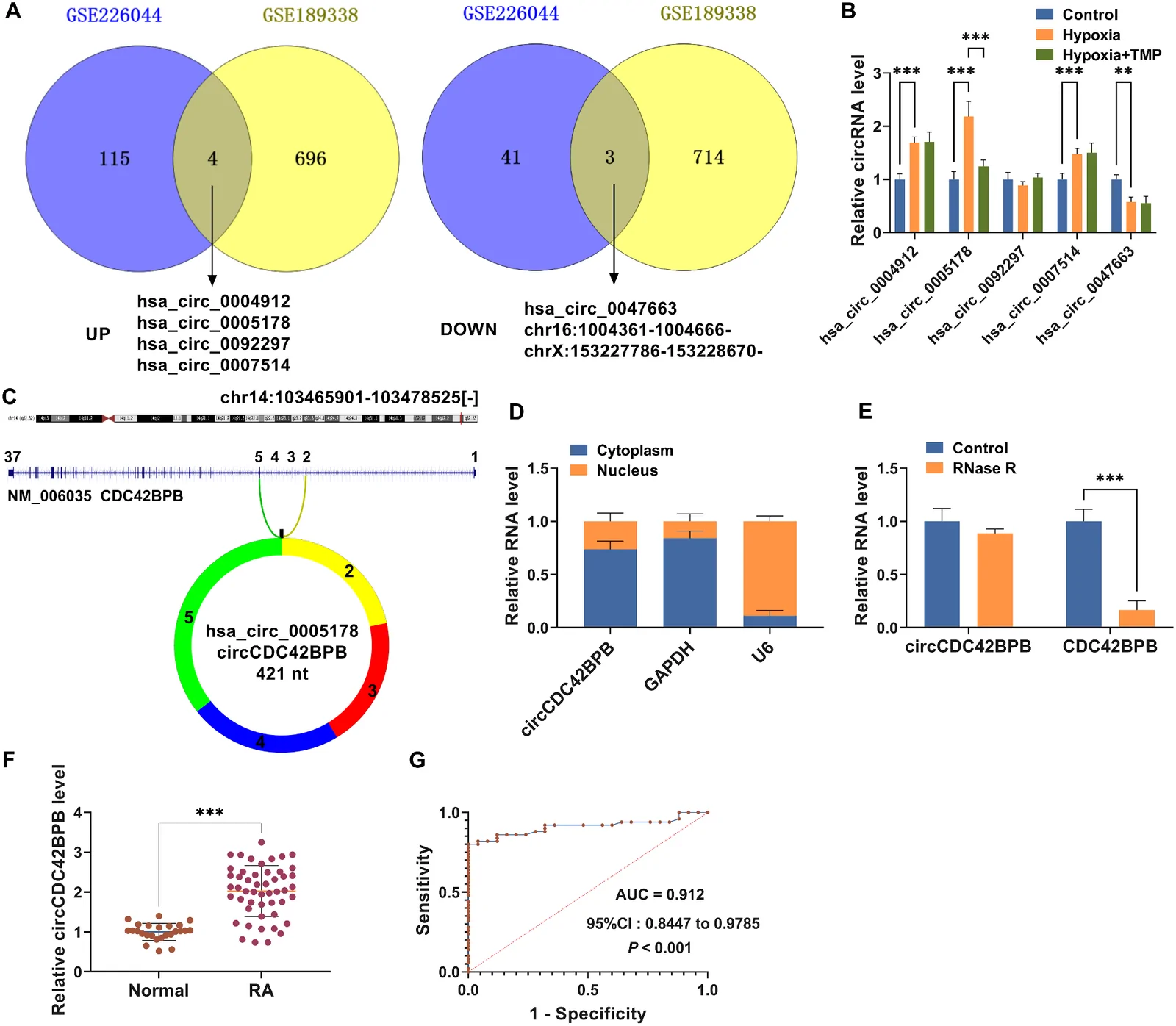
Expression patterns of circCDC42BPB in TMP-treated RA-FLS under hypoxic conditions. (**A**) Left, the Venn diagram is the circRNA that is co-upregulated by the GSE226044 (blue) screen in RA synovial tissue and the GSE189338 (yellow) screen in peripheral blood mononuclear cells (PBMCs) from RA patients. Right, the Venn diagram is the circRNA that is co-downregulated by the GSE226044 screen in RA synovial tissue and the GSE189338 screen in PBMCs from RA patients. (**B**) Expression of these circRNAs was assessed in RA-FLSs treated with hypoxia, hypoxia + TMP, or without by using RT-qPCR (one-way ANOVA). (**C**) The schematic diagram disclosed the formation of hsa_circ_0005178 (circCDC42BPB) from the CDC42BPB gene. (**D**) The cellular localization of circCDC42BPB in RA-FLSs was analyzed using Subcellular fractionation assay. (**E**) CircCDC42BPB and CDC42BPB expression in RA-FLSs with or without RNase treatment was measured using RT-qPCR (one-way ANOVA). (**F**) RT-qPCR analysis of circCDC42BPB expression in the serum of 25 healthy volunteers and 50 RA patients (Student’s *t*-test). (**G**) ROC curve analysis for the diagnostic value evaluation of circCDC42BPB in RA patients. **P < 0.01, ***P < 0.001, *n* = 3

Furthermore, to screen out the candidate functional circRNAs in RA, two microarray gene-profiling data sets GSE226044 (https://www.ncbi.nlm.nih.gov/geo/query/acc.cgi?acc=GSE226044) and GSE189338 (https://www.ncbi.nlm.nih.gov/geo/geo2r/?acc=GSE189338) from Gene Expression Omnibus were utilized to analyze the circRNA expression profile of RA. As shown in Fig. 3A, there were 4 up-regulation and 3 down-regulation circRNAs in RA synovial tissues and PBMCs from RA patients. Then, these circRNAs were subjected to RT-qPCR analysis in the presence of 40 µM TMP under hypoxic conditions. Among these 7 circRNAs, hsa_circ_0005178 displayed the highest fold change (Fig. 3B). Hence, this circRNA was selected for further study. Simultaneously, hsa_circ_0005178 (circCDC42BPB) was produced by exons 2 and 5 of the CDC42BPB gene, and the end of exon 2 and exon 5 was back-spliced to generate the circular structure (Fig. 3C). Subsequently, Subcellular fractionation assay verified that circCDC42BPB was mainly distributed in the cytoplasm fraction of RA-FLSs similarly to GAPDH, used as the cytoplasmic localization control (Fig. 3D). Moreover, we further determined the stability of circCDC42BPB in RA-FLSs using RNase treatment. As presented in Fig. 3E, circCDC42BPB was resistant to RNase R, while linear CDC42BPB mRNA (the host gene of circCDC42BPB) was significantly reduced. Besides, our data confirmed that circCDC42BPB was highly expressed in the serum of 50 RA patients compared with that in the 25 healthy volunteers (Fig. 3F). Apart from that, ROC curve analysis was used to evaluate the potential of circCDC42BPB as a diagnostic marker for RA sufferers. Data exhibited that the AUC reached 0.912 (95% CI = 0.8447–0.9785) in the serum of circCDC42BPB (Fig. 3G). Overall, these results indicated the involvement of circCDC42BPB in TMP-mediated RA-FLSs under hypoxic conditions.

### Overexpression of circCDC42BPB might partially abrogate the repression of TMP on hypoxia-evoked RA-FLS damage

**Fig. 4:**
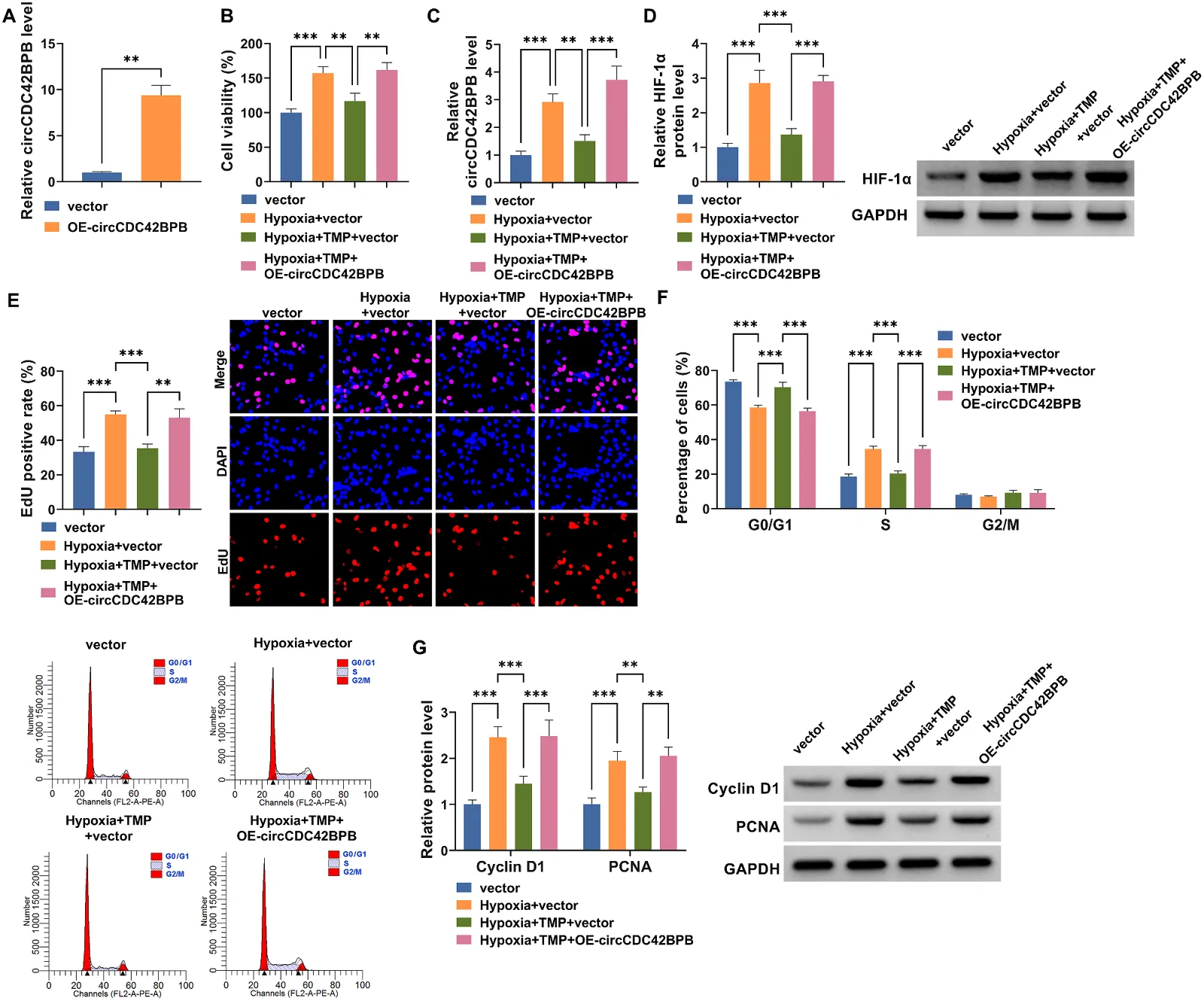
TMP might inhibit hypoxia-caused RA-FLS proliferation via regulating circCDC42BPB. (**A**) The overexpression efficiency of circCDC42BPB in RA-FLSs was determined using RT-qPCR (Student’s *t*-test). (**B**-**E**) RA-FLSs were treated with vector, hypoxia + vector, hypoxia + TMP + vector, or hypoxia + TMP + OE-circCDC42BPB. (**B**) CCK-8 assay was carried out to examine RA-FLS viability (one-way ANOVA). (**C**) CDC42BPB level was detected using RT-qPCR in RA-FLSs treated with vector, hypoxia + vector, hypoxia + TMP + vector, or hypoxia + TMP + OE-circCDC42BPB (one-way ANOVA). (**D**) HIF-1α protein level was determined using western blot in RA-FLSs treated with vector, hypoxia + vector, hypoxia + TMP + vector, or hypoxia + TMP + OE-circCDC42BPB (one-way ANOVA). (**E**) EdU assay was used to examine RA-FLS proliferation (one-way ANOVA). (**F**) Flow cytometry assay was conducted to measure RA-FLS cycle progression (one-way ANOVA). (**G**) Western blot analysis of Cyclin D1 and PCNA protein levels (one-way ANOVA). **P < 0.01, ***P < 0.001, *n* = 3

**Fig. 5:**
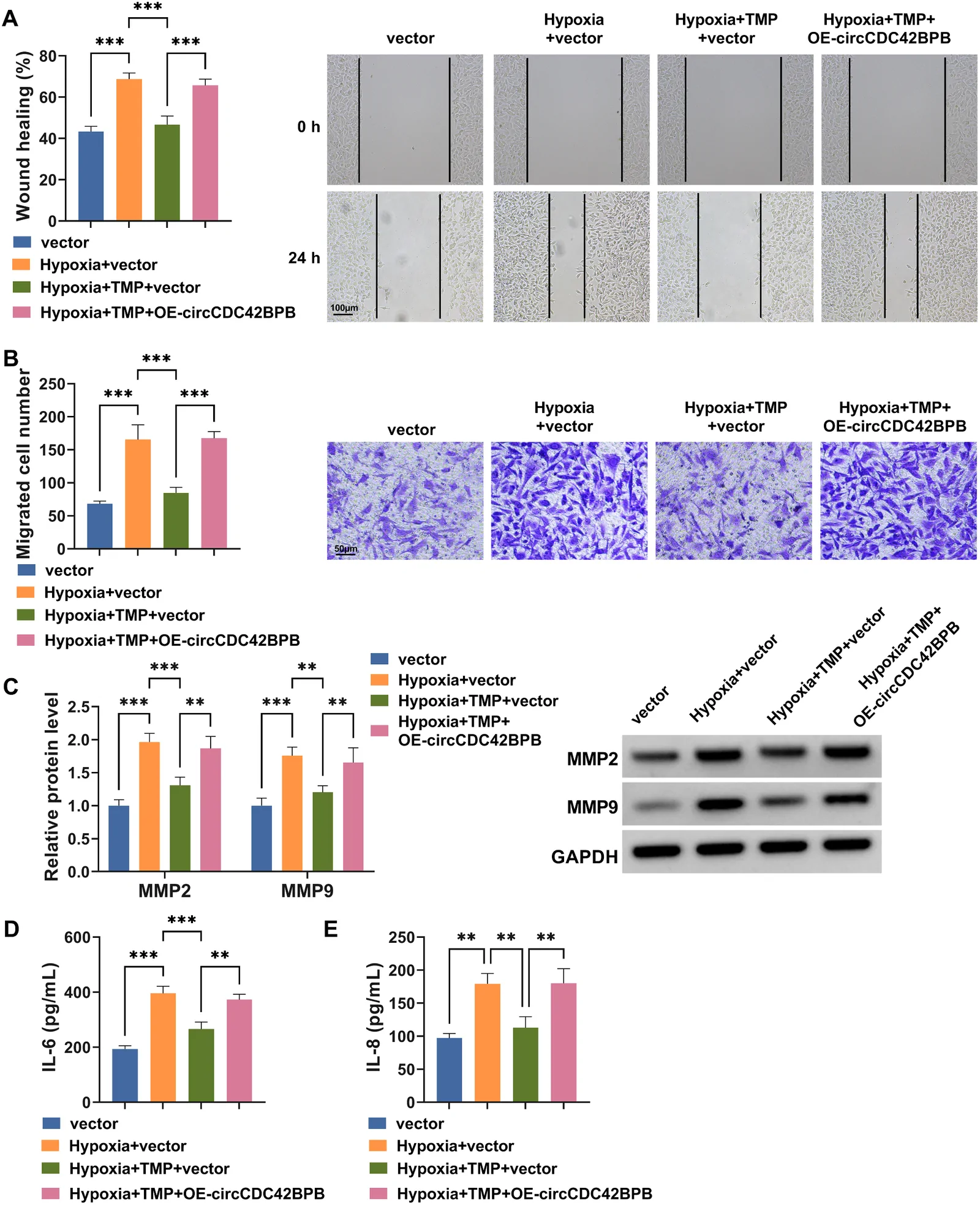
Hypoxia-induced RA-FLS migration and inflammatory response were regulated by TMP/circCDC42BPB. RA-FLSs were treated with vector, hypoxia + vector, hypoxia + TMP + vector, or hypoxia + TMP + OE-circCDC42BPB. (**A** and **B**) RA-FLS migration was monitored by wound healing and transwell assays (one-way ANOVA). (**C**) MMP2 and MMP9 protein levels were tested using western blot in treated RA-FLSs (one-way ANOVA). (**D**) ELISA kits were applied to measure IL-6 and IL-8 secretions in RA-FLSs (one-way ANOVA). **P < 0.01, ***P < 0.001, *n* = 3

Considering that circCDC42BPB presented the lowest expression in TMP-treated RA-FLSs under hypoxic conditions, whether the effects of TMP on RA-FLS development were correlative with circCDC42BPB was further investigated. At first, RT-qPCR results showed that the circCDC42BPB level was significantly elevated in OE-circCDC42BPB-transfected RA-FLSs relative to the control group (Fig. 4A), implying the overexpression efficiency was available for the subsequent research. Functionally, applying TMP might remarkably restrain hypoxia-triggered RA-FLS viability, which was partly abolished after OE-circCDC42BPB introduction (Fig. 4B). Furthermore, our data verified that the introduction of OE-circCDC42BPB might partly abolish the repression of TMP on circCDC42BPB in hypoxia-treated RA-FLSs (Fig. 4C). In addition, we found that the upregulation of circCDC42BPB might clearly overturn the inhibitory effect of TMP treatment on HIF-1α protein level in hypoxia-induced RA-FLSs (Fig. 4D). In parallel, the inhibitory effect of TMP treatment on hypoxia-triggered RA-FLS proliferation and cycle progression was partly abolished via circCDC42BPB overexpression (Fig. 4E and F), as depicted by increased Cyclin D1 and PCNA (Fig. 4G). Moreover, wound healing and transwell assay presented that TMP might strikingly block hypoxia-induced RA-FLS migration, which was partially attenuated through circCDC42BPB upregulation (Fig. 5A and B). Consistently, western blot analysis showed that hypoxia-mediated MMP2 and MMP9 protein level elevation in RA-FLSs was significantly blocked after TMP exposure, whereas this phenomenon was reversed by circCDC42BPB overexpression (Fig. 5C). In addition, hypoxia-evoked inflammatory response in RA-FLSs was alleviated by applying TMP, and this protection was overturned by increased circCDC42BPB, as evidenced by improved IL-6 and IL-8 secretions (Fig. 5D). Besides, our data verified that the overexpression of circCDC42BPB might aggravate hypoxia-triggered RA-FLS proliferation, migration, and inflammatory response (Figure S1). In total, the above-mentioned results elucidated that TMP might dampen hypoxia-induced RA-FLS damage via modulating circCDC42BPB.

### TMP might block HIF-1α-induced circCDC42BPB transcription under hypoxic conditions

**Fig. 6:**
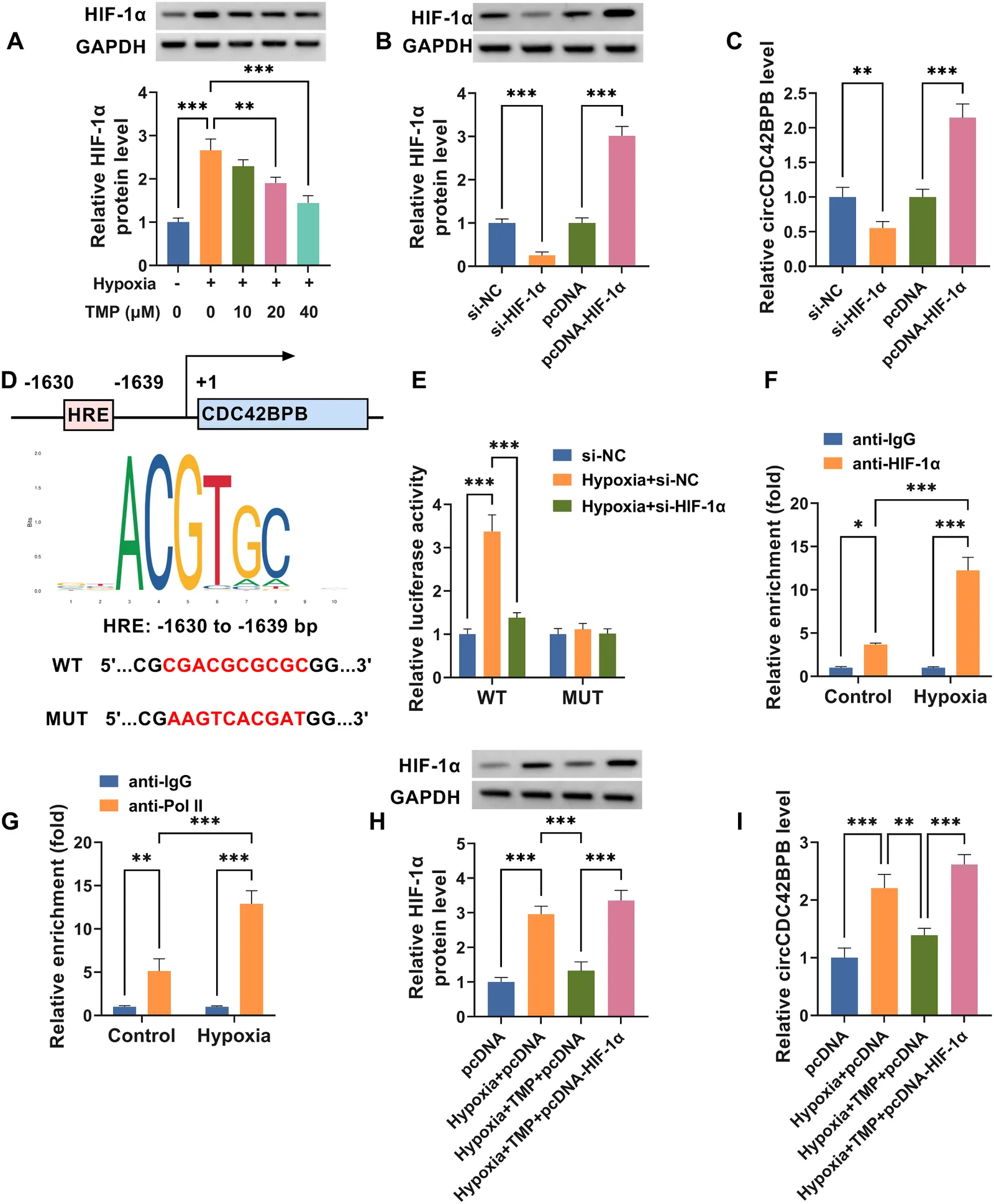
Influence of TMP on HIF-1α-induced circCDC42BPB transcription under hypoxic conditions. (**A**) HIF-1α protein level was determined in RA-FLSs treated with the indicated doses of TMP under hypoxic conditions using western blot (one-way ANOVA). (**B**) Western blot analysis of HIF-1α protein level in RA-FLSs transfected with si-NC, si-HIF-1α, pcDNA, or pcDNA-HIF-1α (one-way ANOVA). (**C**) Effects of HIF-1α knockdown or upregulation on circCDC42BPB expression in RA-FLSs were detected using RT-qPCR (one-way ANOVA). (**D**) Upper schematic represents host gene CDC42BPB HREs obtained from the JASPAR database. Bottom represents that dual-luciferase reporters were constructed with putative CDC42BP HREs and matched mutant HREs in the CDC42BP promoter region. (**E**) A dual-luciferase reporter assay was employed to detect the binding between HIF-1α and the HREs of the circCDC42BPB promoter (one-way ANOVA). (**F**) ChIP assay was performed to assess the binding between HIF-1α and the HREs of the circCDC42BPB promoter under normoxic or hypoxic conditions (one-way ANOVA). (**G**) ChIP analysis of the binding between Pol II and circCDC42BPB promoter under normoxic or hypoxic conditions (one-way ANOVA). (**H** and **I**) HIF-1α protein level and circCDC42BPB expression were respectively measured using western blot and RT-qPCR in RA-FLSs transfected with pcDNA, hypoxia + pcDNA, hypoxia + TMP + pcDNA, or hypoxia + TMP + pcDNA-HIF-1α (one-way ANOVA). *P < 0.05, **P < 0.01, ***P < 0.001, *n* = 3

Additionally, TCMSP and STITCH databases have exhibited that TMP interacts with HIF-1α, which is the master regulator of cellular adaption to hypoxia [35]. Furthermore, recent literature has suggested that HIF-1α, a transcription factor, might promote the transcription of various circRNA parent genes, thereby improving circRNA expression [31]. Therefore, we reasonably speculated that TMP might increase circCDC42BPB expression via regulating HIF-1α under hypoxic conditions. First of all, western blot analysis discovered that HIF-1α protein level RA-FLSs was apparently enhanced hypoxic conditions, which was gradually decreased after TMP treatment in a doses-dependent manner (Fig. 6A). Besides, the knockdown or overexpression of the efficiency of HIF-1α was measured and exhibited in RA-FLSs (Fig. 6B). Furthermore, RT-qPCR results presented that circCDC42BPB content was clearly reduced caused by HIF-1α absence and obviously reinforced via HIF-1α upregulation in RA-FLSs (Fig. 6C). According to the promoter sequence analysis tool JASPAR, possible hypoxia-responsive elements (HREs) were validated in the host gene of circCDC42BPB promoter regions (Fig. 6D). To confirm the direct interaction, a dual-luciferase reporter assay was carried out in RA-FLSs. As shown in Fig. 6E, after hypoxia incubation, the promoter activity of circCDC42BPB was greatly repressed in WT after HIF-1α silencing, rather than MUT-transfected RA-FLSs. Beyond that, ChIP assay conducted on the predicted HREs upstream of the host gene for the circCDC42BPB promoter area indicated that HIF-1α might directly bind to the CDC42BPB promoter region and increase the transcription of the hose gene CDC42BPB during hypoxia (Fig. 6F). Meanwhile, ChIP assay also demonstrated that the binding between RNA polymerase II (Pol II) and the host gene CDC42BPB promoter site was improved during hypoxia, further validating that circCDC42BPB might be activated during hypoxia (Fig. 6G). In addition, western blot results displayed that the co-transfection of pcDNA-HIF-1α might partially counteract the suppressive role of TMP on the protein level of HIF-1α in RA-FLSs under hypoxic conditions (Fig. 6H). Meanwhile, our data proved that applying TMP might evidently hinder circCDC42BPB expression in RA-FLSs during hypoxia, whereas the influence was partially overturned by HIF-1α overexpression (Fig. 6I). In summary, these data illuminated that circCDC42BPB transcription might be regulated by TMP/ HIF-1α under hypoxic conditions.

## Discussion

Recently, many studies have indicated that alterations in tissue oxygen tension contribute to a number of pathologies, including RA. In this disease, synovial expansion is thought to exceed the oxygen supply, thereby producing synovial hypoxia and hypoperfusion [24]. Furthermore, synovial hypoxia might trigger inflammatory RA-FLSs to activate their invasion mechanisms, accelerating RA-FLS proliferation and enhancing migration [30]. Hence, hypoxia is now reasonably postulated to be an important micro-environmental characteristic of RA. Overwhelming evidence suggests that various natural products from TCM have been popularly used in health care and becoming more accepted all over the world [36, 37]. TMP, the active component of the Chinese medicine Ligusticum wallichii Franch (Chuanqiong), has been exhibited to safely inhibit pro-inflammatory pathways and regulate inflammation-related disease [38, 39]. Beyond that, it has been reported that applying TMP might protect against hypoxia-caused oxidative damage in some literature [40, 41]. In the chapter, TMP treatment might hinder hypoxia-triggered RA-FLS proliferation, migration, and inflammatory response in a concentration-dependent way. Apart from that, it is well known that RA-FLSs might synthesize and secrete MMPs, thus leading to the erosion of bone and cartilage [42]. Meanwhile, hypoxia might boost synovial cell migration via increasing MMP-2 and MMP-9 levels [28]. In addition, TMP has also been shown to repress the expression of MMPs in certain diseases, containing arthritis [43]. In this study, the hypoxia-induced MMP-2 and MMP-9 content of RA-FLSs was partially attenuated by applying TMP in a dose-dependent manner. Together, these data provided the first evidence about a possible mechanism for TMP-mediated protection against RA.

Lately, it has become apparent that the emergence of non-coding RNA brings about new light in the investigation of TCM mechanisms. In terms of circRNAs, the continuous advancement of bioinformatics tools and deep sequencing techniques has allowed researchers to identify more interesting facts about circRNAs in various human tissues [44, 45]. Several kinds of literature have discovered that plentiful circRNAs might exert important and powerful regulators in multiple biological activities [46]. Additionally, as a novel player in hypoxia-induced non-coding RNA transcriptomics, hypoxia-responsive circRNAs might regulate the hypoxic response and expedite the progression of hypoxic diseases [47, 48]. In RA, the current work detected the circRNA abnormally expressed using circRNA microarray. Among those candidate functional circRNAs, only circCDC42BPB in hypoxic RA-FLSs was significantly decreased after TMP treatment. Furthermore, our data identified circCDC42BPB as an obviously increased circRNA in RA patients. More importantly, gain-of-function experiments revealed that the overexpression of circCDC42BPB might abolish TMP-caused RA-FLS proliferation, migration, and inflammatory response inhibition under hypoxic conditions. The above findings supported that TMP might suppress hypoxia-induced RA-FLS injury via modulating circCDC42BPB.

As a vital transcription factor in initiating the hypoxic cellular response, HIF-1α has been validated to be widely expressed in rheumatoid synovial tissue and FLSs [25]. In the paper, our results verified that the HIF-1α level was enhanced in RA-FLSs under hypoxic conditions, which was overturned after TMP treatment. Of note, recent studies have reported an interesting mechanism for the regulation of HIF-1α, which might improve the transcription of different circRNA parent genes, thereby affecting human disease progression [31, 49]. HIF-1α could bind to the HREs contained in the promoter region of hypoxia response genes, and hypoxia might increase the affinity of this complex, thus boosting the transcription of hypoxia genes [50]. In this work, the promoter region of circCDC42BPB has a putative HRE. Our data further proved the binding between HIF-1α and HRE of circCDC42BPB, which was fostered under hypoxic conditions. Moreover, HIF-1α silencing might partly abrogate hypoxia-induced intracellular circCDC42BPB content enhancement RA-FLSs. Additionally, rescue experiments validated that elevated HIF-1α might effectively reverse TMP-mediated circCDC42BPB expression repression in RA-FLSs under hypoxic conditions, supporting the regulatory role of the TMP/HIF-1α/circCDC42BPB axis.

## Conclusion

In summary, these data outlined that applying TMP might attenuate hypoxia-induced RA-FLS damage by modulating the HIF-1α/circCDC42BPB, which may contribute to the development of an available preclinical basis for RA treatment.

## Electronic supplementary material

Below is the link to the electronic supplementary material.

**Supplementary Material 1: Figure S1.** Effects of circCDC42BPB overexpression on hypoxia-triggered RA-FLS injury. RA-FLSs were treated with vector, hypoxia+vector, or hypoxia+OE-circCDC42BPB. (**A** and **B**) RA-FLS viability and proliferation were detected using CCK-8 and EdU assays (one-way ANOVA). (**C** and **D**) RA-FLS migration was measured using wound healing and transwell assays (one-way ANOVA). (**E** and **F**) ELISA kits analyzed IL-6 and IL-8 secretions in RA-FLSs (one-way ANOVA). *P <0.05, **P <0.01, ***P <0.001, n = 3

## Data Availability

The analyzed data sets generated during the present study are available from the corresponding author on reasonable request.
